# Identifying Subspace Gene Clusters from Microarray Data Using Low-Rank Representation

**DOI:** 10.1371/journal.pone.0059377

**Published:** 2013-03-19

**Authors:** Yan Cui, Chun-Hou Zheng, Jian Yang

**Affiliations:** 1 School of Computer Science and Technology, Nanjing University of Science and Technology, Nanjing, Jiangsu, China; 2 College of Electrical Engineering and Automation, Anhui University, Hefei, Anhui, China; University of Turin, Italy

## Abstract

Identifying subspace gene clusters from the gene expression data is useful for discovering novel functional gene interactions. In this paper, we propose to use low-rank representation (LRR) to identify the subspace gene clusters from microarray data. LRR seeks the lowest-rank representation among all the candidates that can represent the genes as linear combinations of the bases in the dataset. The clusters can be extracted based on the block diagonal representation matrix obtained using LRR, and they can well capture the intrinsic patterns of genes with similar functions. Meanwhile, the parameter of LRR can balance the effect of noise so that the method is capable of extracting useful information from the data with high level of background noise. Compared with traditional methods, our approach can identify genes with similar functions yet without similar expression profiles. Also, it could assign one gene into different clusters. Moreover, our method is robust to the noise and can identify more biologically relevant gene clusters. When applied to three public datasets, the results show that the LRR based method is superior to existing methods for identifying subspace gene clusters.

## Introduction

With the advent of the DNA microarray technology, it is now possible to study the transcriptional response of a complete genome to different experimental conditions. However, reconstruction the regulatory networks from the high throughput DNA microarray data is one of the foremost challenges of current bioinformatics research. Fortunately, many studies have unveiled that regulatory networks are modular and hierarchically organized [1]–[7]. Since subspace gene clusters may represent co-regulated genes to some degree, so we can reconstruct the whole regulatory network start with identifying the clusters. In addition, since genes can be clustered with similar cellular functions, therefore, identifying the clusters from DNA microarray data might provide much deeper insight into biological function and relevance.

**Figure 1:**
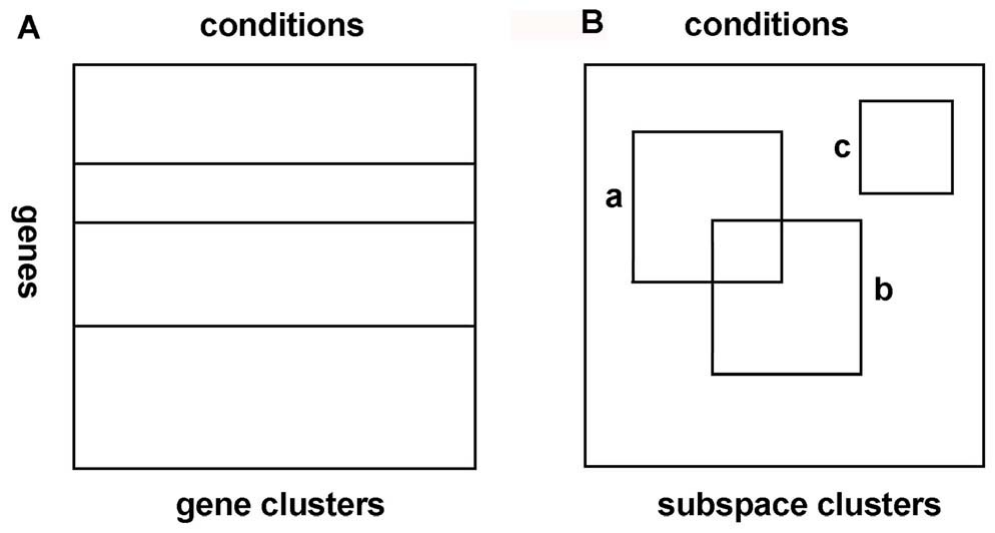
Clustering and subspace clustering of a gene expression matrix: (A) a gene cluster must contain all columns, (B) subspace clusters correspond to arbitrary subsets of rows and columns, shown here as rectangles.

Traditional clustering methods, such as hierarchical clustering [8], *K*-means clustering [9], self-organizing maps [10], and model-based methods [11]–[14] can organize gene expression data into clusters of genes possessing similar expression profiles using all the conditions, and the clusters are exclusive and exhaustive (Figure 1 (A)). These methods identify gene clusters by assuming that genes with similar expression profiles share similar functions or the same pathway. Although these clustering methods classify genes successfully when applied to relatively small data sets, when used for analyzing large-scale expression data, it is limited by three well-recognized drawbacks [3]. Firstly, commonly used algorithms assign each gene to a single cluster, whereas in fact many genes may participate in several functions and should thus be included in several clusters [3], [10]. Secondly, these algorithms group genes on the basis of their expression under all experimental conditions, whereas cellular processes are generally affected only by a small subset of these conditions, so that a gene can participate in multiple clusters or in none at all [15]. In the analysis of a particular cellular process, therefore, most conditions do not contribute information but instead increase the amount of background noise [3]. Thirdly, due to the complex procedures of microarray experiments, gene expression data often contains a huge amount of noise. These algorithms force each gene into a cluster, which may cause the algorithm to be sensitive to noise [15]–[17].

Recently, subspace clustering methods have been proposed to find subgroups of genes that exhibit similar behavior across subsets of samples, experimental conditions, or time points [15], [18]. Subspace clustering was first proposed by Agrawal et al. in general data mining domain [19] to find subsets of objects such that the objects appear as a cluster in a subspace formed by a subset of features. Figure 1 (B) shows an example of the subspace clusters (a and b) embedded in a gene expression matrix. From Figure 1 (B), we can found that a subspace cluster is defined as a submatrix spanned by a set of genes and a set of samples. Genes or samples can be part of more than one subspace cluster or of no subspace cluster. In addition, the subsets of samples for various subspace clusters can be different. Two subspace clusters can share some common genes and samples, and some genes may not belong to any subspace cluster [15]. Therefore, the goal of subspace clustering technique is to find a set of significant subspace clusters in a matrix. Until now, subspace clustering approaches have found widespread applications in many fields [20], e.g., pattern recognition, data compression, image processing and bioinformatics. These methods include algebraic methods [20], such as Generalized Principal Component Analysis(GPCA) [21], iterative methods [20], statistical methods [20], and spectral clustering-based methods [20].

GPCA presents an algebraic way to model the data drawn from a union of multiple subspaces. The intersections between subspaces are automatically allowed; hence, GPCA can deal with both independent and dependent subspaces. However, this method is sensitive to noise and outliers due to the difficulty of estimating the polynomials from real data, which also causes the high computation cost of GPCA [20].

Algebraic algorithms are sensitive to the noise. A very simple way of improving the performance of algebraic algorithms in the case of noisy data is to use iterative refinement. The main advantage of algebraic method is its simplicity since it alternates between assigning points to subspaces and estimating the subspaces via PCA. While the algorithm’s convergence to the global optimum depends on a good initialization, and the method is sensitive to outliers.

Statistical methods include Mixture of Probabilistic PCA (MPPCA) [22], Agglomerative Lossy Compression (ALC) [23] and Random Sample Consensus (RANSAC) [24]. MPPCA is a simple and intuitive method, where each iteration can be computed in closed form by using PPCA. An important drawback of MMPCA is that the number and dimensions of the subspaces need to be known beforehand. In principle, ALC does not need to know the number of subspaces and their dimensions. In practice, however, the number of subspaces is directly related to the parameter of ALC. When the number of subspaces is known, one can run ALC for several values of its parameter. This typically increases the computational complexity of the method. Another disadvantage of ALC, perhaps the major one, is that there is no theoretical proof for the optimality of the agglomerative procedure. The main advantage of RANSAC is its ability to handle outliers explicitly. An important drawback of RANSAC is that its performance deteriorates quickly as the number of subspaces increase. Another critical drawback of RANSAC is that it requires the dimension of the subspaces to be known and equal.

Spectral clustering algorithms are very popular techniques for clustering high-dimensional data. One of the main challenges in applying spectral clustering to the subspace clustering problem is to define a good affinity matrix. This is because two points could be very close to each other but lie in different subspaces (*e.g.*, near the intersection of two subspaces). Conversely, two points could be far from each other but lie in the same subspace. One of the most popular spectral clustering algorithms is sparse subspace clustering (SSC) [25], [26]. SSC is robust to noise and its computational complexity dose not grow exponentially with the number of subspaces and their dimensions. Nonetheless, it requires solving *N* optimization problems in *O*(*N*) variables, hence, it may be time consuming. Another possible disadvantage of SSC is that it is provably correct only in the case of independent or disjoint subspaces.

In this paper, we propose to use Low-Rank Representation(LRR) to identify the subspace gene clusters from microarray data. Compared with traditional techniques, such as *K*-means clustering, our method can cluster genes with similar functions but without similar expression profiles. Moreover, LRR is better fitted to analyze the microarray data than other subspace clustering algorithms, such as GPCA, since it is robust to noise and outliers via lowest-rank criterion, and it also can be capable of extracting useful information from a high level of background noise. The contribution of the paper is that a new subspace gene clustering method based on LRR is proposed, and its theoretical analysis is also given.

## Materials and Methods

### Low-Rank Representation

Before we present the Low-Rank Representation(LRR) based method for identifying gene clusters from microarray data, we first introduce the algorithm of Low-Rank Representation, which is a new framework for seeking the lowest rank representation matrix [27]. Supposing that there is a gene expression dataset with 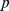 genes and 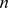 samples, we can denote it as a matrix 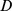 with size 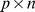. When the data is noiseless, the LRR algorithm looks for a representation 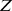 by solving the problem
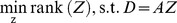
(1)


We call the optimal solutions 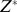 of the above problem the “lowest-rank representation” of the data 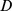 with respect to a dictionary 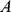. The above optimization problem is difficult to solve due to the discrete nature of the rank function. Fortunately, as suggested by matrix completion methods [28], [29], the following convex optimization provides a good surrogate for problem(1):
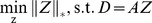
(2)where 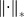 denotes the nuclear norm of a matrix [27], [30], *i.e.*, the sum of the singular values of the matrix. Note that the block diagonal structure of 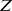 directly induces clustering genes (each block corresponds to a cluster). So the clustering task is equivalent to finding a block diagonal representation matrix 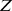.

However, due to the complex procedures of microarray experiments, gene expression data often contains a huge amount of noise. Therefore, the optimization model of LRR is formulated as:
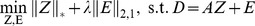
(3)where 
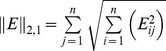
 is the 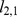-norm of the matrix of errors 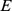. Minimizing the 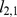-norm of noise is to meet the assumption that some data vectors are corrupted and others are clean. Since in this case, the solution 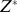 to Eq.(3) may not be block diagonal, it is recognized as an affinity matrix instead and spectral clustering methods are applied to 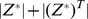 to obtain a block diagonal matrix, where 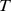 denotes the matrix or vector transpose and 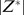 denotes a matrix whose entries are the absolute values of 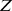.

The LRR algorithm proceeds by solving the optimization problem in (3) using an Augmented Lagrange Multiplier (ALM) method [31]–[33].

### Identifying Gene Clusters Using LRR

Denoted the gene expression data matrix as 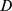 with size 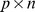, each row of 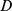 containing the expression levels of a gene in all the 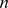 samples, and each column of 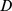 containing the expression levels of all the 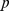 genes in one sample. Our goal of using LRR algorithm to model the gene expression data is to discover subspace gene clusters, so we can cluster the genes according to their representation matrices. When we apply the LRR algorithm to cluster genes, we need to use 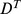 instead of 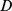. The Eq.(3) can be written as:
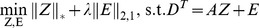
(4)


In order to cluster the genes into their respective subspaces, we need to compute an affinity matrix that encodes the pairwise affinities between data vectors. So we use the data 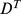 itself as the dictionary, i.e., problem(4) becomes
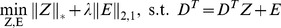
(5)


Then Eq.(5) can be written as
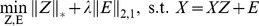
(6)where 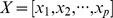 (each column is a gene), 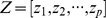 is the coefficient matrix with each 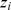 being the representation of 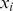. 
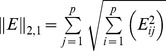
 is 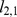-norm, and the parameter 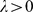 is used to balance the effects of the two parts, which could be chosen according to properties of the two norms, or tuned empirically. Since 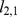-norm encourages the columns of 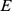 to be zero, the underlying assumption here is that some data vectors are corrupted and the others are clean.

The optimization problem (6) is convex and can be solved by various methods [31]. For efficiency, we adopt in this paper the Augmented Lagrange Multiplier(ALM) method [31]–[33]. We first convert Eq.(6) to the following equivalent problem:
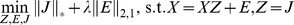
(7)


This problem can be solved by the ALM method, which minimizes the following augmented Lagrange function:
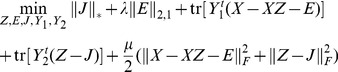
(8)


The above problem is unconstrained. So it can be minimized with respect to 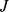, *Z* and *E*, respectively, by fixing the other variables, and then updating the Lagrange multipliers 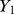 and 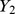, where 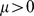 is a penalty parameter. The inexact ALM method, also called the alternating direction method, is outlined in Algorithm 1. Its convergence properties could be proven similarly as those in [32].

After solving problem (6), as in [25], we utilize the lowest-rank representation (denoted by 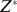) to define the affinity matrix of an undirected graph. We treat gene clustering problem as a graph partitioning problem. The gene vectors correspond to the vertices and the affinity between 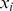 and 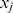 is computed by 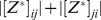. In clustering, we seek to partition the set of vertices into disjoint sets where by some measure the similarity among the vertices in a set is high and across different sets is low. So we could use the spectral clustering algorithms such as Normalized Cuts [34] to produce the final clustering results, since Normalized Cuts is an unbiased measure which can minimize the disassociation between the clusters of a graph and maximize the association within the clusters simultaneously. The final clusters are gene clusters that we discover from the gene expression data. Integrating LRR with spectral clustering has the following advantages. First, since LRR may fail to obtain a block-diagonal representation in complex applications, the spectral clustering could ensure the robustness of the clustering. Second, it is convenient to integrate the lowest-rank representation with other information by defining such an undirected graph For example, in some specific applications such as subspace segmentation, ones may want to enforce that only the neighbor samples can be connected by edges. Therefore, the proposed clustering method based LRR can be capable of extracting useful information from a huge amount of background noise and it also can well capture the intrinsic patterns of genes with similar functions. Algorithm 2 summarizes the whole clustering algorithm of LRR for gene expression data.

**Algorithm 1** Solving Problem (6) by Inexact ALM

**Input:** data matrix *X*, parameter 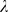


**Initialized:** 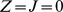, 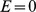, 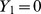, 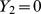, 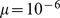,


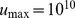
, 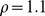, 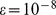.

**while** not converged **do**

**1)** fix the others and update *J* by
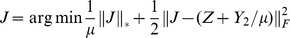



**2)** fix the others and update *Z* by
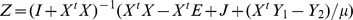



**3)** fix the others and update *E* by
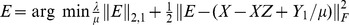



**4)** update the multipliers
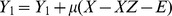


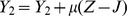



**5)** update the parameter 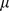 by 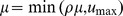


**6)** check the convergence conditions
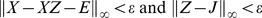



**end while**

**Algorithm 2** Subspace Clustering by LRR

**Input:** data matrix 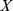, number of subspaces 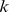


**1)** Obtain the lowest-rank representation by solving problem(6)

**2)** Construct an undirected graph by using the lowest-rank

representation to define the affinity matrix of the graph

**3)** Use Normalized Cuts to cluster the vertices of the graph into 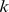 clusters

### Gene Expression Data

In this paper, three published gene expression datasets are used to analyze the performance of our methods: the yeast dataset [35], the yeast_Spellman dataset [36] and nomal human tissue dataset [37].

The yeast dataset contains 173 samples collected under several different conditions, which include temperature shocks, hyper-and hypoosmotic shocks, exposure to various agents such as peroxide, menadione, diamide, dithiothreitol, amino acid starvation, nitrogen source depletion and progression into stationary phase, etc. This dataset contains 6152 genes in each sample.

The yeast_Spellman data contains 6178 genes in 73 samples under several different conditions. There are different carbon sources, temperature, yeast strain background, etc.

The normal human tissue data consists of Affymetrix oligonucletide array measurements of 7070 genes in 59 samples of 19 kinds of tissues.

### GeneCodis Analysis

GeneCodis is a web-based tool for finding sets of biological annotations that frequently appear together and are significant in a set of genes. It allows the integrated analysis of annotations from different sources and generates statistical rank scores for signal annotations and their combinations. GeneCodis is an important extension of existing tools for the functional analysis of genes lists [38]–[40], and it is publicly available at http://genecodis.cnb.csic.es.

The application of GeneCodis is simple. It takes a list of genes which are in a cluster as input and determines biological annotations or combinations of annotations that are over-represented with respect to a reference list. Meanwhile, selecting one or more categories that you want include in the analysis is necessary. In addition, the organism selected is Saccharomyces cerevisiae for yeast dataset and yeast_Spellman data, while the organism selected is Homo sapiens for normal human tissue dataset. When the genes are submitted, the modular enrichment analysis and singular enrichment analysis can be obtained. For a detailed description of this method, see the online help for the program.

In the GeneCodis method, *P*-values obtained through Hypergeometric analysis corrected by false discovery rate (FDR) method [41], [42]. Briefly, a gene list of the same size of the input list is generated by randomly selecting genes from the set of genes defined as the reference distribution. The process of extracting frequent sets of annotations is repeated and *P*-values for the annotations and combinations of annotations generated from this random list are calculated using the same statistical test. This process is repeated 100 times and the corrected *P*-values for each set of K-annotations are calculated as the fraction of permutations having any annotation of the same value of K with a *P*-value as good or better than the observed *P*-value.

## Results and Discussion

In this section, the proposed method is applied to identify the subspace gene clusters in the form of functional links among genes. The GeneCodis is executed to investigate the enrichment of functional annotations of genes in each cluster. First, LRR based methods and *K*-means, GPCA are carried on the synthetic data. Then, these methods are used to find gene clusters from real gene expression data. The MATLAB code is supplied in File S1 and File S2.

### Simulation on Synthetic Data

**Figure 2:**
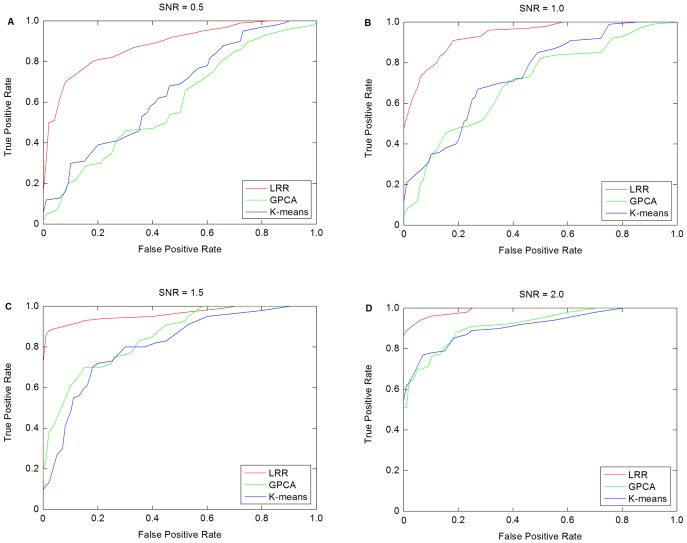
ROC curves for synthetic data. (SNR denotes the signal-to-noise ratio).

We first tested our method on synthetic data to assess its performance for clustering. We synthesize a network that consists of 2000 genes each with 200 samples and 20 transcription factors. We construct 20 independent subspaces 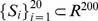 whose bases 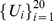 are computed by 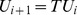, 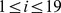, where 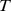 is a random rotation and 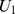 is a random orthogonal matrix of dimension 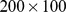. So, each subspace has a dimension of 100. We sample 100 data vectors from each subspace by 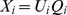, 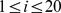 with 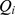 being a 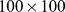 noise matrix, 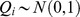. Then the noise matrix is added to data matrix with different Signal-to-Noise Ratios (SNR). The simulation scheme in [27] is used to generate the synthetic data. The average receiver operator characteristic (ROC) curves are shown in Figure 2 with four different SNR (signal-to-noise ratio). The corresponding 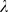 in LRR based method are set as 0.01, 0.1, 1 and 10, respectively.

From Figure 2, we can see that for different SNRs, our LRR based method consistently outperforms the competitive methods. When the noise level increases (*i.e.*, SNR decreases), three methods suffer from performance degradation by a corresponding decrease in the AUCs (Table 1). However, the AUC range of LRR is from 0.9908 to 0.8928, while the AUC ranges of *K*-means and GPCA are from 0.9233 and 0.9255 to 0.6643 and 0.6154, respectively. The decrease rates of *K*-means and GPCA are higher than that of LRR. In other word, *K*-means and GPCA are sensitive to the noise, while LRR is robust to the noise. Moreover, the proposed method can extract useful information from a high level of background noise.

**Table 1:** AUC statistics for synthetic data.

	SNR = 0.5	SNR = 1.0	SNR = 1.5	SNR = 2.0
*K*-means	0.6643	0.7547	0.8253	0.9233
GPCA	0.6145	0.7128	0.8652	0.9255
LRR	0.8928	0.9435	0.9681	0.9908

From the experiments on synthetic data, a conclusion can be drawn that LRR method outperforms other methods for clustering.

### Experimental Results on the Yeast Dataset

For yeast dataset, we first used KNNimpute [43] to fill in missing values. When we apply our method to cluster the genes, the parameters 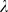 and *k* need to be considered. Since the original gene expression matrix obtained from a scanning process contains noise, missing values, and systematic variations arising from the experimental procedure. The gene expression data contains a huge amount of noise. In our method, the parameter 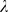 is used to balance the effects of noise. For the yeast dataset, we take 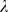 = 0.1, because when taking this value, the enrichment analysis based on GO can achieve the most significant result. With regards to the parameter *k*, more clusters are discovered with the increase of *k*. In this experiment, according to the former work [44]–[46], we choose *k* = 30.

**Figure 3:**
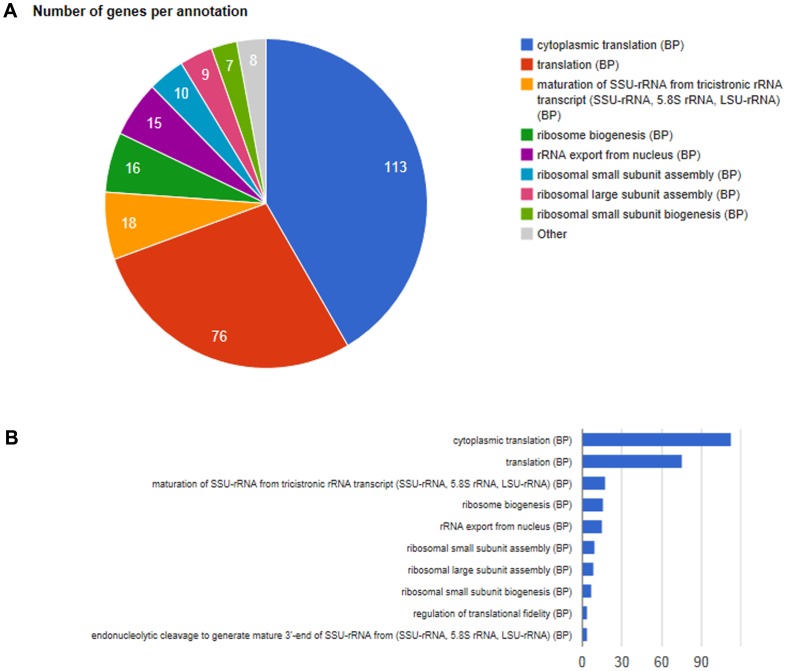
Enriched combinations of significant annotations of Biological Process of Cluster C17: (A) pie graph, (B) bar graph.

**Figure 4:**
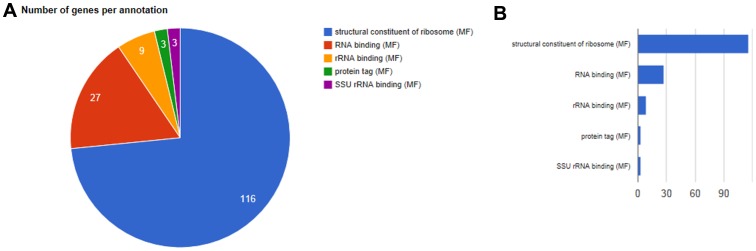
Enriched combinations of significant annotations of Molecular Function of Cluster C17: (A) pie graph, (B) bar graph.

Table 2 lists the most enriched GO categories of modular enrichment analysis in each cluster uncovered from the yeast dataset. In this table, the first two columns list total number of genes in each cluster and the number of annotated genes in each cluster respectively. It should be noted that among the 6152 genes, only 6123 genes were annotated by GO and KEGG database. The last column on the right are *P*-values obtained through Hypergeometric analysis corrected by false discovery rate (FDR) method. In Table 3, we analyzed the function of genes clustered in two clusters. For example, there are 14 out of 30 genes clustered sharing similar function ‘response to stress’, and Cluster C3 is significantly associated with ‘response to stress’. Therefore, genes share with similar function can be clustered in the same cluster. Table 4 lists a few functional categories in each significantly enriched modules (corrected *P*-value <10^−20^). Figures 3 and 4 show the enriched combinations of significant annotations of Biological Process (BP) of C17 and that of Molecular Function of C17 respectively, using pie graphs and bar graphs.

**Table 2:** The most enriched GO categories of modular enrichment in each gene clusters uncovered by LRR from yeast dataset.

Cluster	No. of genes with in functional category	Major GO categories	Corrected *P*-value
C1(121genes)	10	Starch and sucrose metabolism	2.99342E-11
C2(86genes)	4	structural constituent of cytoskeleton	4.7389E-2
C3(30genes)	14	response to stress	6.33965E-30
C4(663genes)	151	integral to membrane	3.67556E-2
C5(45genes)	27	oxidation-reduction process	1.04843E-25
C6(38genes)	3	DNA repair	4.92582E-5
C7(69genes)	10	ion transport	6.84207E-13
C8(71genes)	16	Glycolysis/Gluconeogenesis	3.15299E-19
C9(181genes)	92	ribosome biogenesis	9.62533E-119
C10(87genes)	5	prospore membrane	2.30387E-5
C11(34genes)	10	helicase activity	9.29836E-11
C12(414genes)	10	hydrolase activity	2.44361E-5
C13(551genes)	11	regulation of transcription, DNA-dependent	2.0876E-3
C14(114genes)	37	cellular amino acid biosynthetic process	2.33136E-41
C15(393genes)	3	mitotic recombination	1.67731E-4
C16(25genes)	20	transposition, RNA-mediated	3.23626E-35
C17(130genes)	116	structural constituent of ribosome	3.58803E-202
C18(454genes)	77	Biosynthesis of secondary metabolites	1.57577E-32
C19(83genes)	3	sporulation resulting in formation of a cellular spore	2.12806E-2
C20(511genes)	3	oxidation-reduction process	4.34938E-4
C21(75genes)	3	transferase activity, transferring phosphorus-containing groups	9.06387E-3
C22(27genes)	3	metal ion binding	1.04585E-5
C23(675genes)	5	transport	5.76795E-3
C24(183genes)	92	ribisome biogenesis	3.82873E-112
C25(553genes)	73	transcription, DNA-dependent	2.91171E-7
C26(287genes)	35	extracellular region	2.62718E-24
C27(50genes)	31	mitochondrion	2.79651E-53
C28(801genes)	38	vesicle-mediated transport	1.40759E-8
C29(347genes)	5	guanyl-nucleotide exchage factor activity	1.6044E-4
C30(258genes)	30	fungal-type cell wall	1.30198E-22

The columns of the table summarize the total sizes of the module (numbers in parentheses), the number of genes annotated in the cluster, the GO categories associated with the cluster, and the *P*-value after FDR correction.

**Table 3:** Results of genes analysis in gene clusters uncovered by LRR from yeast dataset.

Cluster	Major GOcategories	Genes
C3 (14/30)	response to stress	YBL075C,YBR082C,YBR169C,YDR171W,YDR214W,YDR258C,YER103W,YFL016C,YJL034W,YJR045C,YLL024C,YLL026W, YLR259C,YMR186W
C16 (20/25)	transposition,RNA-mediated	YAR009C,YBL005W-A,YBR012W-A,YBR012WB, YCL019W,YCL020W,YER138C,YER160C,YHR214CB, YJR026W,YJR027W,YJR028W,YJR029W,YML039W,YML040W,YML045W,YMR045C,YMR046C,YMR050C,YMR051C

Only selected two enriched functional categories and the corresponding annotated genes are presented. The columns of the table summarize the number of annotated genes in the module versus the total size of the cluster (numbers in the parentheses), the GO categories associated with the cluster, and a set of annotated genes.

**Table 4:** Singular enrichment of GO (or KEGG) categories in gene clusters uncovered by LRR from yeast dataset.

Cluster	NG	Corrected *P*-value	Annotations
C3	17	1.22794E-23	protein folding (BP)
	15	2.25951E-20	unfolded protein binding (MF)
	3	2.89824E-5	TRC complex (CC)
	11	5.73217E-14	Protein processing in endoplasmic reticulum (KEGG)
C5	25	3.27373E-25	oxidation-reduction process (BP)
	27	1.23536E-25	oxidoreductase activity (MF)
	4	2.51171E-3	mitochondrial intermembrane space (CC)
	5	1.79973E-8	Linoleic acid metabolism (KEGG)
C9	92	5.76648E-107	ribosome biogenesis (BP)
	13	1.13044E-15	snoRNA binding (MF)
	113	3.1653E-108	nucleolus (CC)
	26	7.46338E-20	ribosome biogenesis in eukaryotes (KEGG)
C14	37	6.24985E-41	cellular amino acid biosynthetic process (BP)
	30	2.12287E-10	catalytic activity (MF)
	2	4.07894E-3	sulfite reductase complex (NADPH) (CC)
	32	3.31541E-20	Biosynthesis of secondary metabolites (KEGG)
C16	20	9.50421E-34	transposition,RNA-mediated (BP)
	12	5.27074E-20	ribonuclease H activity (MF)
	20	1.38697E-34	retrotransposon nucleocapsid (CC)
C17	113	3.08315E-199	cytoplasmic translation (BP)
	116	2.51791E-169	structural constituent of ribosome (MF)
	67	5.19679E-102	cytosolic large ribosomal subunit (CC)
	116	1.16868E-198	Ribosome (KEGG)
C18	54	8.94213E-11	oxidation-reduction process (BP)
	69	2.07793E-11	catalytic activity (MF)
	215	7.75913E-15	plasma membrane enriched fraction (CC)
	77	6.06951E-34	Biosynthesis of secondary metabolites (KEGG)
C24	92	3.82873E-112	ribisome biogenesis (BP)
	13	1.33056E-15	snoRNA binding (MF)
	113	2.07758E-107	nucleolus (CC)
	26	9.9672E-20	Ribosome biogenesis in eukaryotes (KEGG)
C26	31	7.8069E-19	cellular cell wall organization (BP)
	11	8.43025E-8	cyclin-dependent protein kinase regulator activity (MF)
	35	4.84777E-25	fungal-type cell wall (CC)
	26	1.1831E-10	Cell cycle-yeast (KEGG)
C27	10	1.40896E-16	ATP synthesis coupled proton transport (BP)
	10	8.57624E-14	proton-transporting ATPase activity, rotational mechanism (MF)
	31	1.69019E-34	mitochondrial inner membrane (CC)
	31	1.42892E-50	Oxidative phosphorylation (KEGG)
C30	50	4.52988E-17	cell cycle (BP)
	10	5.70948E-7	cyclin-dependent protein kinase regulator activity (MF)
	30	2.94031E-22	fungal-type cell wall (CC)
	26	9.06875E-12	Cell cycle-yeast (KEGG)

Only significantly enriched functional categories (corrected *P*-value<10^−20^) are presented. The columns of the table summarize the total sizes of the cluster (numbers in parentheses), the number of annotated genes in the cluster, the *P*-value after FDR correction, and the GO categories associated with the cluster.

We also adopted *K*-means clustering and GPCA as compared methods applying on the yeast dataset, and the experimental results are listed in Tables 5 and 6. These two tables show *K*-means clustering and GPCA are efficient in clustering the genes. However, our method can identify more significantly enriched clusters, *e.g.* 11 clusters were discovered with corrected *P*-value <10^−20^, while only 4 and 1 clusters by *K*-means and GPCA, respectively. The number of annotated genes in each cluster using our algorithm is more than that of other two methods.

**Table 5:** The most enriched categories of modular enrichment in each gene clusters uncovered by *K*-means clustering from yeast dataset.

Cluster	No. of genes with infunctional category	Major GO categories	Corrected *P*-value
C1(133genes)	39	regulation of cyclin-dependent protein	1.4052E-11
C2(259genes)	54	regulation of transcription, DNA-dependent	2.05448E-10
C3(259genes)	57	DNA binding	2.72567E-13
C4(327genes)	11	Peroxisome	4.10943E-6
C5(219genes)	11	protein targeting to ER	3.96874E-5
C6(131genes)	33	regulation of transcription, DNA-dependent	2.1956E-8
C7(216genes)	17	nucleotide binding	4.16395E-4
C8(152genes)	22	protein folding	1.68904E-15
C9(193genes)	5	ATP binding	2.8324E-4
C10(203genes)	18	hydrolase activity	1.07105E-13
C11(396genes)	131	translation	7.02517E-134
C12(171genes)	3	nucleic acid binding	3.46107E-3
C13(152genes)	3	nucleosome assembly	1.48603E-2
C14(126genes)	30	mitochondrial translation	3.81352E-44
C15(191genes)	12	mRNA processing	9.64789E-6
C16(261genes)	71	membrane	2.59589E-20
C17(131genes)	21	response to stress	2.73901E-9
C18(96genes)	13	cellular amino acid biosynthetic process	5.76184E-16
C19(321genes)	14	cytoplasm	8.42409E-13
C20(130genes)	13	nucleotide binding	2.13419E-8
C21(227genes)	3	membrane	3.85374E-2
C22(154genes)	79	integral to membrane	4.31568E-11
C23(207genes)	51	transcription	2.17224E-13
C24(435genes)	133	Ribosome biogenesis	1.04031E-103
C25(127genes)	59	integral to membrane	2.25528E-4
C26(180genes)	66	integral to membrane	4.56057E-4
C27(277genes)	17	sporulation resulting in formation of a cellular spore	4.20206E-2
C28(165genes)	4	ubiquitin-dependent protein catabolic process	2.09826E-4
C29(123genes)	24	mitochondrion	6.43371E-33
C30(223genes)	12	oxidation-reduction process	6.82702E-4

The columns of the table summarize the total sizes of the cluster (numbers in parentheses), the number of genes annotated in the cluster, the GO categories associated with the cluster, and the *P*-value after FDR correction.

**Table 6:** The most enriched categories of modular enrichment in each gene clusters uncovered by GPCA from yeast dataset.

Cluster	No. of genes with infunctional category	Major GO categories	Corrected *P*-value
C1(271genes)	4	oxidation-reduction process	3.05689E-2
C2(194genes)	3	metabolic process	2.5142E-2
C3(214genes)	5	ribosome biogenesis	1.92418E-3
C4(234genes)	17	regulation of transcription, DNA-dependent	4.2973E-4
C5(203genes)	17	transposition, RNA-mediated	1.61358E-7
C6(207genes)	6	proteolysis	5.36602E-6
C7(194genes)	3	metal ion binding	2.00179E-5
C8(228genes)	5	DNA replication	7.49636E-5
C9(200genes)	7	catalytic activity	5.24079E-5
C10(173genes)	20	cytoplasmic translation	6.84959E-11
C11(219genes)	3	ubiquitin-protein ligase activity	2.88482E-5
C12(205genes)	6	glycolysis	1.42749E-8
C13(210genes)	5	protein refolding	4.29455E-7
C14(183genes)	5	transport	1.77438E-5
C15(224genes)	5	purine base biosynthetic process	1.73835E-7
C16(235genes)	4	phosphorylation	1.65757E-5
C17(200genes)	7	ATP binding	5.24079E-5
C18(89genes)	3	DNA repair	1.89736E-6
C19(203genes)	7	structural constituent of ribosome	5.25787E-7
C20(189genes)	4	flavin adenine dinucleotide binding	1.10535E-3
C21(215genes)	3	mitotic spindle elongation	1.06714E-4
C22(185genes)	3	sequence-specific DNA binding	1.66864E-4
C23(197genes)	52	ribosome biogenesis	1.69208E-32
C24(202genes)	9	nucleosome assembly	5.30185E-13
C25(190genes)	17	rRNA processing	7.47168E-7
C26(168genes)	3	small GTPase mediated signal transduction	1.2521E-4
C27(219genes)	36	regulation of transcription, DNA-dependent	6.05907E-9
C28(216genes)	8	cellular aldehyde metabolic process	9.75059E-11
C29(221genes)	8	ergosterol biosynthetic process	8.66254E-10
C30(205genes)	28	structural constituent of ribosome	6.60734E-14

The columns of the table summarize the total sizes of the cluster(numbers in parentheses), the number of genes annotated in the cluster, the GO categories associated with the cluster, and the *P*-value after FDR correction.

**Figure 5:**
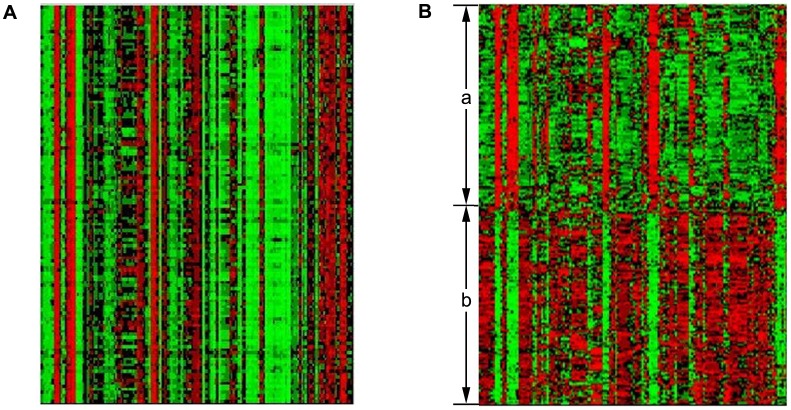
Two heatmaps of expression values of genes analyzed by the proposed algorithm from the yeast dataset: (A) a heatmap of expression values of genes in Cluster C17, and the heatmap shows similar expression patterns of genes in different samples, (B) a heatmap of expression values of genes in Cluster C14, and the heatmap shows different expression patterns of genes in different samples (denoted as a and b).

Moreover, the proposed approach is effective in clustering together genes with similar expression profiles and similar function categories. Cluster C17, for example, is strongly associated with the ‘structural constituent of ribosome’ process (corrected *P*-value <3.58803×10^−202^). The heatmap indicated similar expression patterns of genes under different experimental conditions (Figure 5(A)). More significantly, our algorithm can cluster genes which show different expression profiles but similar functions. Cluster C14, for example, is significantly enriched by ‘cellular amino acid biosynthetic process’ (corrected P-value <2.33136×10^−41^). The heatmap revealed as least two distinctive expression patterns in this cluster (denoted as a and b, Figure 5(B)). Which show the advantage of our subspace clustering method.

We also compared statistical significance of common enriched functional categories in gene clusters uncovered by our algorithm and *K*-means (Table 7). Among those common functional categories detected significantly by these methods, there are five out of eight functional categories that our method produced significantly lower corrected *P*-value than *K*-means method did. In addition, our method is robust to the noise, while other two methods are not. We also found the performance of GPCA is not better than that of *K*-means clustering for this dataset. To investigate the performance of these methods explicitly, Table 8 lists the average values of negative logarithm of corrected *P*-value on three datasets using *K*-means, GPCA and LRR, respectively. From Table 8 (a), we also can see that LRR based method outperforms other methods on yeast dataset.

**Table 7:** Comparison of statistical significance of enriched functional categories in gene clusters uncovered by LRR and *K*-means from yeast dataset.

Major GO categories	LRR	*K*-means
hydrolase activity	2.44361E-5 (10/414)	1.07105E-13 (18/203)
response to stress	6.33965E-30 (14/30)	2.73901E-9 (21/131)
cellular amino acid biosynthetic process	2.33136E-41 (37/114)	5.76184E-16 (13/96)
integral to membrane	3.67556E-2 (151/663)	4.56057E-4 (66/180)
sporulation resulting in formation of a cellular spore	2.12806E-2 (3/83)	4.20206E-2 (17/277)
mitochondrion	2.79651E-53 (31/50)	6.43371E-33 (24/123)
oxidation-reduction process	1.04843E-25 (27/45)	6.82702E-4 (12/223)
regulation of transcription, DNA-dependent	2.0876E-3 (11/551)	2.05448E-10 (54/259)

Only selected common significantly enriched functional categories are presented. The columns of the table summarize the GO categories associated with the cluster, the *P*-values after FDR correction by each approach, and the number of genes in the cluster that are annotated with the corresponding GO category versus the total size of the cluster(numbers in the parentheses).

**Table 8:** The average values of negative logarithm of corrected *P*-value on three datasets.

	a	b	c
	Yeast Dataset	Yeast_Spellman Dataset	Normal Human Tissue Dataset
*K*-means	17.0343	10.7687	6.6664
GPCA	6.7035	5.3445	9.7273
LRR	27.0948	13.5402	20.1414

In the table, (a), (b) and (c) list the average values of negative logarithm of corrected *P*-value on Yeast Dataset, Yeast_Spellman dataset and Normal Human Tissue Dataset using three methods, respectively.

### Experimental Results on Yeast_Spellman Dataset

**Figure 6:**
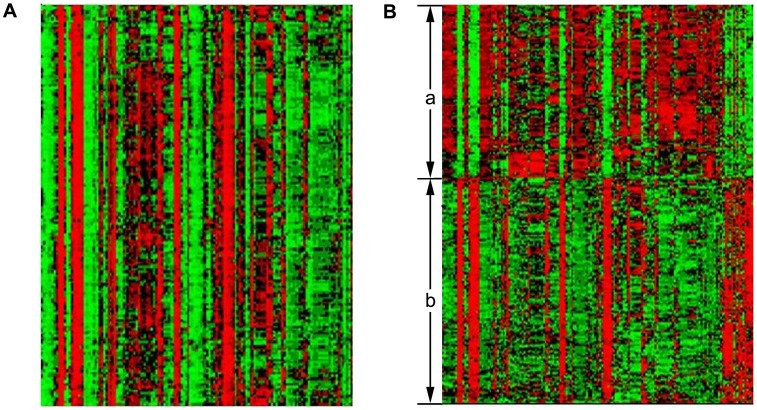
Two heatmaps of expression values of genes analyzed by the proposed algorithm from the yeast_Spellman dataset: (A) a heatmap of expression values of genes in Cluster C27, and the heatmap shows similar expression patterns of genes in different samples, (B) a heatmap of expression values of genes in Cluster C10, and the heatmap shows different expression patterns of genes in different samples (denoted as a and b).

We also first used KNNimpute to fill in missing values. In this experiment, we chose 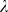 = 0.01 due to the dataset contains a huge amount of noise. Table S1 (In File S1 and File S2) lists the most enriched GO categories of modular enrichment analysis in each gene cluster uncovered from the yeast_Spellman dataset. In this dataset, there are 6074 genes of 6178 genes annotated by GO and KEGG. Table S2 also lists a few functional categories in each significantly enriched modules (corrected *P*-value <10^−10^). Figures 6(A) and Figure 6(B) demonstrate that the proposed method can identify the clusters of genes with similar expression profiles or different expression profiles, respectively. Cluster C27 significantly enriched by ‘ribosome biogenesis’ (corrected *P*-value <2.47852×10^−104^), and Cluster C10 is significantly enriched by ‘structural constituent of ribosome’ (corrected *P*-value <2.63854×10^−38^). Compared with experimental results of *K*-means clustering and GPCA methods listed in Tables S3 and S4, our approach can discover 11 significantly enriched clusters with corrected *P*-value <10^−10^, while 8 and 3 clusters by *K*-means clustering and GPCA, respectively. We also compared statistical significance of common enriched functional categories in gene clusters uncovered by our algorithm and *K*-means (Table S5). Among those common functional categories detected significantly by these methods, there are six out of eight functional categories that our method produced significantly lower corrected *P*-value than *K*-means method did. In the experiment we also found that the result of GPCA is not robust to noise. In addition, for yeast_Spellman dataset, the average value of negative logarithm of corrected *P*-value using LRR based method listed in Table 8 (b) is higher than the values listed in Table 8 (b) using *K*-means and GPCA, respectively. Therefore, LRR based method can achieve better result than other methods.

### Experimental Results on Normal Human Tissue Dataset

**Figure 7:**
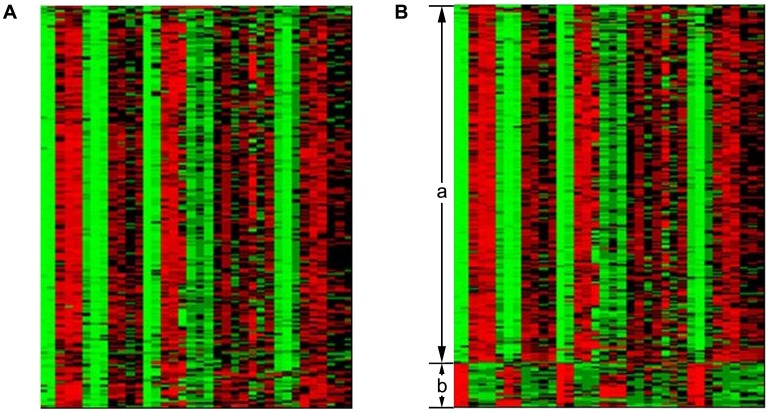
Two heatmaps of expression values of genes analyzed by the proposed algorithm from the normal human tissue dataset: (A) a heatmap of expression values of genes in Cluster C18, and the heatmap shows similar expression patterns of genes in different samples, (B) a heatmap of expression values of genes in Cluster C3, and the heatmap shows different expression patterns of genes in different samples (denoted as a and b).

In this experiment, we chose 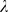 = 10 since the dataset contains some noise but no missing values. Table S6 lists the most enriched GO categories of modular enrichment analysis in each gene clusters discovered from the human tissue dataset, and only 5991 genes were annotated by GO and KEGG among the 7070 genes. Among 30 gene clusters, ten clusters were significantly enriched by GO and KEGG (corrected *P*-value <10^−20^). Each cluster appeared to be dominated by only a few functional categories (Table S7). Similar to above two experiments, Figure 7(A) shows the heatmap of Cluster C18, which is significantly enriched by ‘muscle filament sliding’ (corrected *P*-value <1.29274×10^−39^). It can be seen that the expression patterns of genes in this cluster are similar and similar function categories. On the contrary, the heatmap of Cluster C3, which is significantly enriched by ‘extracellular region’ (corrected *P*-value <4.79992×10^−25^), reveals different expression profiles but similar functions (Figure 7(B)).

The experimental results of the *K*-means clustering and GPCA methods are listed in Tables S8 and S9. Our method can discover 10 significantly enriched clusters with corrected *P*-value <10^−20^, while *K*-means and GPCA can identify 1and 3 clusters, respectively. Moreover, from Table 8 (c), we also can find that the performance of LRR based method is better than that of other methods. Similar to above two experiments, we compared statistical significance of common enriched functional categories in gene clusters uncovered by our algorithm and *K*-means (Table S10). Among those common functional categories detected significantly by these methods, there are five functional categories that our method produced significantly all lower corrected *P*-value than *K*-means method did. For this dataset, the performance of GPCA is better than *K*-means clustering.

### Conclusions

In this study, we present low-rank representation based method for identifying subspace gene clusters from microarray data. The new approach can cluster the genes via low-rank criterion. Our goal is to find a block diagonal representation matrix from gene expression data using low-rank representation. In this block diagonal matrix each block corresponds to a cluster. Therefore, the genes in each cluster have similar functions. Compared with other clustering methods, the proposed method offers several advantages. Firstly, it can identify genes of similar functions yet without similar expression profiles. Secondly, the method can assign one gene into different modules. Thirdly, our method is capable of extracting useful information from a high level of background noise. In a word, our method leads to a significant improvement in identifying biologically relevant gene clusters. In the experiment we also found that many categories discovered by different methods are different. So in practice, different methods can be used to find more reliable result.

Otherwise, subspace gene clusters identified using the proposed method may represent co-regulated genes to some degree. However, due to the limited information present in any dataset, genes in the same cluster might be co-expressed but not necessarily co-regulated [47]–[49]. Therefore, to design an effective algorithm for finding co-regulated genes is our future work.

## Supporting Information

Table S1 **The most enriched GO categories of modular enrichment in each gene clusters uncovered by LRR from yeast_Spellman dataset.**
(DOC)

Table S2 **Singular enrichment of GO (or KEGG) categories in gene clusters uncovered by LRR from yeast_Spellman dataset.**
(DOC)

Table S3 **The most enriched categories of modular enrichment in each gene clusters uncovered by** ***K*** **-means clustering from yeast_Spellman dataset.**
(DOC)

Table S4 **The most enriched categories of modular enrichment in each gene clusters uncovered by GPCA from yeast_Spellman dataset.**
(DOC)

Table S5 **Comparison of statistical significance of enriched functional categories in gene clusters uncovered by LRR and** ***K*** **-means from yeast_Spellman dataset.**
(DOC)

Table S6 **The most enriched GO categories of modular enrichment in each gene clusters uncovered by LRR from normal human tissue dataset.**
(DOC)

Table S7 **Singular enrichment of GO (or KEGG) categories in gene clusters uncovered by LRR from normal human tissue dataset.**
(DOC)

Table S8 **The most enriched categories of modular enrichment in each gene clusters uncovered by K-means clustering from normal human tissue dataset.**
(DOC)

Table S9 **The most enriched categories of modular enrichment in each gene clusters uncovered by GPCA from normal human tissue dataset.**
(DOC)

Table S10 **Comparison of statistical significance of enriched functional categories in gene clusters uncovered by LRR and** ***K*** **-means from normal human tissue dataset.**
(DOC)

File S1 **The MATLAB code of Low-Rank Representation is supplied in this Supplementary File.**
(DOC)

File S2 **The yeast dataset used in our experiment is supplied in this Supplementary File.**
(XLS)
