# Supplementary material for: Identifying Subspace Gene Clusters from Microarray Data Using Low-Rank Representation
Source: PLoS One. 2013 Mar 19;8(3):e59377. doi: 10.1371/journal.pone.0059377 (PMC3602020; doi:10.1371/journal.pone.0059377)
Supplement: Table S1 — The most enriched GO categories of modular enrichment in each gene clusters uncovered by LRR from yeast_Spellman dataset. (DOC) [file pone.0059377.s001.doc]

Table S1. The most enriched GO categories of modular enrichment in each gene clusters uncovered by LRR from yeast_Spellman dataset.

| **Cluster** | **No. of genes with in functional category** | **Major GO categories** | **Corrected *P*-value** |
| --- | --- | --- | --- |
| C1(56genes) | 4 | mitochondrial translation | 1.892E-4 |
| C2(163genes) | 11 | plasma membrane enriched fraction | 7.43455E-8 |
| C3(63genes) | 39 | cytoplasmic translation | 1.51206E-51 |
| C4(183genes) | 15 | response to stress | 3.21632E-17 |
| C5(226genes) | 43 | oxidation-reduction process | 2.93288E-16 |
| C6(115genes) | 3 | hydrolase activity | 2.11076E-4 |
| C7(306genes) | 4 | nucleotide binding | 3.36876E-6 |
| C8(114genes) | 10 | telomere maintenance via recombination | 1.7592E-11 |
| C9(221genes) | 6 | transport | 5.20336E-6 |
| C10(39genes) | 28 | structural constituent of ribosome | 2.63854E-38 |
| C11(32genes) | 11 | extracellular region | 1.01513E-12 |
| C12(144genes) | 3 | transport | 1.9991E-2 |
| C13(94genes) | 3 | catalytic activity | 8.87207E-6 |
| C14(163genes) | 3 | mRNA processing | 4.65532E-5 |
| C15(1010genes) | 102 | endoplasmic reticulum | 1.21403E-12 |
| C16(137genes) | 6 | metal ion binding | 5.55649E-5 |
| C17(215genes) | 38 | translation | 1.51665E-32 |
| C18(141genes) | 3 | mRNA processing | 1.46235E-4 |
| C19(83genes) | 5 | structural constituent of ribosome | 2.22023E-3 |
| C20(255genes) | 4 | nuclear envelope lumen | 1.61818E-6 |
| C21(143genes) | 5 | endocytosis | 3.47137E-6 |
| C22(68genes) | 6 | karyogamy involved in conjugation with cellular fusion | 2.14656E-7 |
| C23(355genes) | 68 | transport | 1.78484E-5 |
| C24(79genes) | 3 | Protein processing in endoplasmic reticulum | 7.10697E-6 |
| C25(747genes) | 5 | transferase activity | 1.26574E-5 |
| C26(121genes) | 3 | hydrolase activity | 9.26798E-7 |
| C27(204genes) | 86 | ribosome biogenesis | 2.47852E-104 |
| C28(70genes) | 9 | nucleosome assembly | 5.43046E-16 |
| C29(117genes) | 12 | DNA replication | 2.85007E-12 |
| C30(56genes) | 3 | zinc ion binding | 9.41855E-4 |
| The columns of the table summarize the total sizes of the cluster (numbers in parentheses), the number of genes annotated in the cluster, the GO categories associated with the cluster, and the *P*-value after FDR correction. | | | |
