# Supplementary material for: Identifying Subspace Gene Clusters from Microarray Data Using Low-Rank Representation
Source: PLoS One. 2013 Mar 19;8(3):e59377. doi: 10.1371/journal.pone.0059377 (PMC3602020; doi:10.1371/journal.pone.0059377)
Supplement: Table S2 — Singular enrichment of GO (or KEGG) categories in gene clusters uncovered by LRR from yeast_Spellman dataset. (DOC) [file pone.0059377.s002.doc]

Table S2. Singular enrichment of GO (or KEGG) categories in gene clusters uncovered by LRR from yeast_Spellman dataset.

| **Cluster** | **NG** | **Corrected *P*-value** | **Annotations** |
| --- | --- | --- | --- |
| C3 | 39 | 7.54324E-47 | cytoplasmic translation (BP) |
|  | 40 | 2.20747E-43 | structural constituent of ribosome (MF) |
|  | 33 | 2.10364E-29 | ribosome (CC) |
|  | 40 | 5.1297E-51 | ribosome (KEGG) |
| C4 | 23 | 3.55784E-14 | protein folding (BP) |
|  | 20 | 4.31745E-12 | unfolded protein binding (MF) |
|  | 68 | 8.63041E-11 | mitochondrion (CC) |
|  | 12 | 3.42375E-5 | Protein processing in endoplasmic reticulum (KEGG) |
| C5 | 43 | 7.74916E-16 | oxidation-reduction process (BP) |
|  | 43 | 1.90662E-16 | oxidoreductase activity (MF) |
|  | 84 | 1.71139E-13 | mitochondrion (CC) |
|  | 29 | 1.4406E-8 | Biosynthesis of secondary metabolites (KEGG) |
| C8 | 10 | 1.20366E-11 | telomere maintenance via recombination (BP) |
|  | 8 | 4.18674E-9 | DNA helicase activity (MF) |
|  | 12 | 1.49823E-4 | cellular bud neck (CC) |
|  | 11 | 8.41971E-12 | DNA replication (KEGG) |
| C10 | 27 | 1.59782E-34 | cytoplasmic translation (BP) |
|  | 28 | 4.29842E-33 | structural constituent of ribosome (MF) |
|  | 15 | 5.09198E-18 | cytosolic large ribosomal subunit (CC) |
|  | 28 | 1.4224E-38 | Ribosome (KEGG) |
| C11 | 6 | 1.19795E-10 | cytokinesis, completion of separation (BP) |
|  | 3 | 1.38108E-3 | structural constituent of cell wall (MF) |
|  | 10 | 1.20439E-12 | cell wall (CC) |
|  | 2 | 4.43298E-2 | Amino sugar and nucleotide sugar metabolism (KEGG) |
| C15 | 21 | 4.14971E-5 | lipid metabolic process (BP) |
|  | 102 | 1.21403E-12 | endoplasmic reticulum (CC) |
| C17 | 45 | 2.32608E-31 | cytoplasmic translation (BP) |
|  | 44 | 1.02385E-21 | structural constituent of ribosome (MF) |
|  | 47 | 1.28086E-21 | ribosome (CC) |
|  | 43 | 2.50824E-28 | Ribosome (KEGG) |
| C27 | 88 | 1.27424E-102 | ribosome biogenesis (BP) |
|  | 42 | 1.38754E-10 | RNA binding (MF) |
|  | 31 | 1.48758E-92 | preribosome, large subunit precursor (CC) |
|  | 26 | 3.65558E-18 | Ribosome biogenesis in eukaryotes (KEGG) |
| C28 | 9 | 1.60087E-12 | nucleosome assembly (BP) |
|  | 2 | 2.84927E-4 | minus-end-directed microtubule motor activity (MF) |
|  | 9 | 7.47838E-16 | nuclear nucleosome (CC) |
|  | 9 | 3.16882E-8 | MAPK signaling pathway-yeast (KEGG) |
| C29 | 10 | 7.9002E-12 | telomere maintenance via recombination (BP) |
|  | 8 | 5.26963E-9 | DNA helicase activity (MF) |
|  | 5 | 6.64543E-5 | replication fork (CC) |
|  | 12 | 2.703E-13 | DNA replication (KEGG) |
| Only significantly enriched functional categories (corrected *P*-value<10^-10^) are presented. The columns of the table summarize the total sizes of the cluster (numbers in parentheses), the number of annotated genes in the cluster, the *P*-value after FDR correction, and the GO categories associated with the cluster. | | | |
