# Supplementary material for: Identifying Subspace Gene Clusters from Microarray Data Using Low-Rank Representation
Source: PLoS One. 2013 Mar 19;8(3):e59377. doi: 10.1371/journal.pone.0059377 (PMC3602020; doi:10.1371/journal.pone.0059377)
Supplement: Table S3 — The most enriched categories of modular enrichment in each gene clusters uncovered by K -means clustering from yeast_Spellman dataset. (DOC) [file pone.0059377.s003.doc]

Table S3. The most enriched categories of modular enrichment in each gene clusters uncovered by *K*-means clustering from yeast_Spellman dataset.

| **Cluster** | **No. of genes with**  **in functional category** | **Major GO categories** | **Corrected *P*-value** |
| --- | --- | --- | --- |
| C1(160genes) | 3 | transcription, DNA-dependent | 2.64138E-3 |
| C2(198genes) | 11 | nucleotide binding | 9.90304E-6 |
| C3(175genes) | 3 | regulation of transcription, DNA-dependent | 3.19278E-3 |
| C4(191genes) | 10 | integral to membrane | 3.93278E-4 |
| C5(165genes) | 81 | mitochondrion | 4.35469E-9 |
| C6(207genes) | 29 | rRNA processing | 2.95715E-20 |
| C7(195genes) | 38 | Biosynthesis of secondary metabolites | 6.97316E-16 |
| C8(225genes) | 22 | membrane | 3.3782E-11 |
| C9(202genes) | 5 | meiosis | 2.62408E-3 |
| C10(202genes) | 34 | mitochondrion | 7.45474E-27 |
| C11(226genes) | 18 | DNA binding | 1.54174E-5 |
| C12(196genes) | 10 | cytoplasm | 1.10362E-4 |
| C13(188genes) | 61 | ribosome | 8.57438E-78 |
| C14(232genes) | 11 | regulation of transcription, DNA-dependent | 4.26091E-3 |
| C15(236genes) | 18 | mRNA processing | 4.82476E-4 |
| C16(184genes) | 74 | membrane | 2.12621E-4 |
| C17(201genes) | 24 | sporulation resulting in formation of a cellular spore | 1.41939E-8 |
| C18(214genes) | 61 | integral to membrane | 1.35519E-3 |
| C19(200genes) | 5 | transport | 2.44646E-4 |
| C20(227genes) | 51 | ribosome biogenesis | 8.57415E-42 |
| C21(229genes) | 14 | DNA replication | 2.01205E-12 |
| C22(205genes) | 4 | transporter activity | 3.58933E-2 |
| C23(195genes) | 6 | oxidation-reduction process | 2.54935E-3 |
| C24(225genes) | 3 | metal ion binding | 8.69519E-4 |
| C25(176genes) | 29 | transport | 1.19374E-3 |
| C26(201genes) | 5 | protein transport | 3.47411E-3 |
| C27(174genes) | 13 | ATP binding | 3.97375E-4 |
| C28(177genes) | 20 | response to stress | 2.00411E-6 |
| C29(242genes) | 5 | endoplasmic reticulum | 2.13871E-4 |
| C30(215genes) | 45 | structural constituent of ribosome | 1.53563E-40 |
| The columns of the table summarize the total sizes of the cluster (numbers in parentheses), the number of genes annotated in the cluster, the GO categories associated with the cluster, and the *P*-value after FDR correction. | | | |
