# Supplementary material for: Identifying Subspace Gene Clusters from Microarray Data Using Low-Rank Representation
Source: PLoS One. 2013 Mar 19;8(3):e59377. doi: 10.1371/journal.pone.0059377 (PMC3602020; doi:10.1371/journal.pone.0059377)
Supplement: Table S4 — The most enriched categories of modular enrichment in each gene clusters uncovered by GPCA from yeast_Spellman dataset. (DOC) [file pone.0059377.s004.doc]

Table S4. The most enriched categories of modular enrichment in each gene clusters uncovered by GPCA from yeast_Spellman dataset.

| **Cluster** | **No. of genes with**  **in functional category** | **Major GO categories** | **Corrected *P*-value** |
| --- | --- | --- | --- |
| C1(160genes) | 4 | metal ion binding | 7.82312E-3 |
| C2(198genes) | 4 | action cytoskeleton organization | 6.694669E-3 |
| C3(175genes) | 13 | fungal-type cell wall | 1.61201E-7 |
| C4(191genes) | 6 | Ribosome biogenesis in eukaryotes | 5.70516E-5 |
| C5(165genes) | 20 | oxidation-reduction process | 2.58369E-3 |
| C6(207genes) | 3 | endoplasmic reticulum | 3.19155E-2 |
| C7(195genes) | 16 | helicase activity | 4.35156E-8 |
| C8(225genes) | 4 | Biosynthesis of secondary metabolites | 4.4878E-4 |
| C9(202genes) | 4 | transport | 1.48665E-2 |
| C10(202genes) | 4 | lagging strand elongtion | 2.9192E-7 |
| C11(226genes) | 18 | Ribosome biogenesis | 7.41293E-6 |
| C12(196genes) | 16 | rRNA processing | 7.20306E-5 |
| C13(188genes) | 16 | ribosome biogenesis | 1.19729E-5 |
| C14232genes) | 5 | vaculoe fusion, non-autophagic | 1.39508E-5 |
| C15(236genes) | 3 | condenssed nuclear chromosome | 3.4383E-4 |
| C16(184genes) | 3 | spindle pole body | 6.69547E-5 |
| C17(201genes) | 9 | nucleosome assembly | 3.3615E-11 |
| C18(214genes) | 34 | structural constituent of ribosome | 5.84542E-19 |
| C19(200genes) | 25 | ribonucleoprotein complex | 2.46882E-17 |
| C20(227genes) | 4 | trehalose biosynthetic process | 3.28599E-5 |
| C21(229genes) | 3 | electron transport chain | 3.14485E-4 |
| C22(205genes) | 4 | glycogen metabolic process | 1.23044E-4 |
| C23(195genes) | 4 | sequence-specific DNA binding transcription factor activity | 4.42784E-4 |
| C24(225genes) | 3 | ascospore wall assembly | 3.12963E-5 |
| C25(176genes) | 23 | cell cycle | 2.64052E-6 |
| C26(201genes) | 4 | transport | 1.13964E-5 |
| C27(174genes) | 4 | plasma membrane enriched fraction | 6.48932E-5 |
| C28(177genes) | 5 | transferase activity | 3.70067E-6 |
| C29(242genes) | 5 | hydrolase activity | 2.25978E-6 |
| C30(215genes) | 12 | vesicle-mediated transport | 4.78863E-5 |
| The columns of the table summarize the total sizes of the cluster (numbers in parentheses), the number of genes annotated in the cluster, the GO categories associated with the cluster, and the *P*-value after FDR correction. | | | |
