# Supplementary material for: Identifying Subspace Gene Clusters from Microarray Data Using Low-Rank Representation
Source: PLoS One. 2013 Mar 19;8(3):e59377. doi: 10.1371/journal.pone.0059377 (PMC3602020; doi:10.1371/journal.pone.0059377)
Supplement: Table S5 — Comparison of statistical significance of enriched functional categories in gene clusters uncovered by LRR and K -means from yeast_Spellman dataset. (DOC) [file pone.0059377.s005.doc]

Table S5. Comparison of statistical significance of enriched functional categories in gene clusters uncovered by LRR and *K*-means from yeast_Spellman dataset.

| **Major GO categories** | **LRR** | ***K*-means** |
| --- | --- | --- |
| nucleotide binding | 3.36876E-6 (4/306) | 9.90304E-6 (11/198) |
| transport | 5.20336E-6 (6/221) | 2.44646E-4 (5/200) |
| ribosome biogenesis | 2.47852E-104 (86/204) | 8.57415E-42 (51/227) |
| DNA replication | 2.85007E-12 (12/117) | 2.01205E-12 (14/229) |
| oxidation-reduction process | 2.93288E-16 (43/226) | 2.54935E-3 (6/195) |
| metal ion binding | 5.55649E-5 (6/137) | 8.69519E-4 (3/225) |
| response to stress | 3.21632E-17 (15/183) | 2.00411E-6 (20/177) |
| structural constituent of ribosome | 2.63854E-38 (28/39) | 1.53563E-40 (45/215) |
| Only selected common significantly enriched functional categories are presented. The columns of the table summarize the GO categories associated with the cluster, the *P*-values after FDR correction by each approach, and the number of genes in the cluster that are annotated with the corresponding GO category versus the total size of the cluster (numbers in the parentheses). | | |
