# Supplementary material for: Identifying Subspace Gene Clusters from Microarray Data Using Low-Rank Representation
Source: PLoS One. 2013 Mar 19;8(3):e59377. doi: 10.1371/journal.pone.0059377 (PMC3602020; doi:10.1371/journal.pone.0059377)
Supplement: Table S6 — The most enriched GO categories of modular enrichment in each gene clusters uncovered by LRR from normal human tissue dataset. (DOC) [file pone.0059377.s006.doc]

Table S6. The most enriched GO categories of modular enrichment in each gene clusters uncovered by LRR from normal human tissue dataset.

| **Cluster** | **No. of genes with**  **in functional category** | **Major GO categories** | **Corrected *P*-value** |
| --- | --- | --- | --- |
| C1(267genes) | 34 | signal transduction | 7.95854E-8 |
| C2(217genes) | 17 | respiratory eletron transport chain | 5.69991E-18 |
| C3(65genes) | 35 | extracellular region | 4.79992E-25 |
| C4(11genes) | 4 | digestion | 3.44689E-10 |
| C5(33genes) | 5 | keratinocyte differentiation | 1.28723E-11 |
| C6(767genes) | 344 | protein binding | 3.75319E-102 |
| C7(90genes) | 14 | extracellular region | 6.95675E-23 |
| C8(141genes) | 25 | sequence-specific DNA binding transcription factor activity | 1.64793E-11 |
| C9(67genes) | 16 | endoplasmic reticulum | 1.05036E-8 |
| C10(303genes) | 115 | protein binding | 2.29766E-24 |
| C11(41genes) | 16 | epidermis development | 1.42368E-30 |
| C12(161genes) | 68 | peptidase activity | 4.48738E-17 |
| C13(212genes) | 82 | protein homodimerization activity | 9.24286E-18 |
| C14(242genes) | 98 | nucleotide binding | 2.83491E-23 |
| C15(412genes) | 63 | signal transduction | 2.25236E-20 |
| C16(314genes) | 55 | receptor activity | 3.84536E-15 |
| C17(104genes) | 55 | plasma membrane | 4.79362E-24 |
| C18(41genes) | 17 | muscle filament sliding | 1.29274E-39 |
| C19(305genes) | 36 | receptor activity | 1.4778E-19 |
| C20(285genes) | 119 | nucleus | 4.54684E-22 |
| C21(202genes) | 21 | soluble fraction | 1.62363E-12 |
| C22(126genes) | 12 | oxidoreductase activity | 8.66324E-13 |
| C23(31genes) | 17 | extracellular region | 2.33134E-12 |
| C24(117genes) | 16 | synaptic transmission | 1.0502E-10 |
| C25(19genes) | 17 | extracellular space | 3.30548E-18 |
| C26(145genes) | 37 | mitochondrion | 4.08274E-17 |
| C27(281genes) | 20 | synaptic transmission | 5.28408E-12 |
| C28(363genes) | 55 | gene expression | 2.89145E-40 |
| C29(168genes) | 13 | regulation of transcription, DNA-dependent | 6.4354E-5 |
| C30(399genes) | 36 | positive regulation of transcription from RNA polymerase II promoter | 2.06584E-13 |
| The columns of the table summarize the total sizes of the cluster (numbers in parentheses), the number of genes annotated in the cluster, the GO categories associated with the cluster, and the *P*-value after FDR correction. | | | |
