# Supplementary material for: Identifying Subspace Gene Clusters from Microarray Data Using Low-Rank Representation
Source: PLoS One. 2013 Mar 19;8(3):e59377. doi: 10.1371/journal.pone.0059377 (PMC3602020; doi:10.1371/journal.pone.0059377)
Supplement: Table S7 — Singular enrichment of GO (or KEGG) categories in gene clusters uncovered by LRR from normal human tissue dataset. (DOC) [file pone.0059377.s007.doc]

Table S7. Singular enrichment of GO (or KEGG) categories in gene clusters uncovered by LRR from normal human tissue dataset.

| **Cluster** | **NG** | **Corrected *P*-value** | **Annotations** |
| --- | --- | --- | --- |
| C3 | 12 | 1.89445E-18 | negative regulation of endopeptidase activity (BP) |
|  | 12 | 4.26257E-17 | serine-type endopeptidase inhibitor activity (MF) |
|  | 35 | 4.79992E-25 | extracellular region (CC) |
|  | 11 | 1.93363E-17 | Complement and coagulation cascades (KEGG) |
| C6 | 56 | 4.35381E-24 | gene expression (BP) |
|  | 344 | 1.60396E-103 | protein binding (MF) |
|  | 198 | 7.57145E-66 | cytosol (CC) |
|  | 41 | 8.15109E-17 | Pathways in cancer (KEGG) |
| C7 | 13 | 1.10246E-7 | blood coagulation (BP) |
|  | 12 | 4.06023E-7 | oxidoreductase activity (MF) |
|  | 22 | 8.20005E-15 | extracellular space (CC) |
|  | 14 | 1.32717E-21 | Complement and coagulation cascades (KEGG) |
| C10 | 25 | 2.4941E-12 | positive regulation of cell proliferation (BP) |
|  | 115 | 2.53802E-25 | protein binding (MF) |
|  | 126 | 2.31522E-24 | nucleus (CC) |
|  | 21 | 2.42136E-12 | MAPK signaling pathway (KEGG) |
| C11 | 16 | 2.37279E-30 | epidermis development (BP) |
|  | 13 | 1.95682E-17 | structural molecule activity (MF) |
|  | 11 | 1.26464E-17 | intermediate filament (CC) |
|  | 2 | 3.23122E-2 | Tyrosine metabolism (KEGG) |
| C14 | 35 | 9.94357E-10 | signal transduction (BP) |
|  | 98 | 5.06233E-24 | nucleotide binding (MF) |
|  | 66 | 2.08177E-11 | plasma membrane (CC) |
|  | 14 | 1.19933E-6 | Neuroactive ligand-receptor interaction (KEGG) |
| C17 | 22 | 1.72003E-17 | blood coagulation (BP) |
|  | 25 | 5.52942E-10 | antigen binding (MF) |
|  | 55 | 6.33959E-25 | plasma membrane (CC) |
|  | 10 | 5.634E-10 | Natural killer cell mediated cytotoxicity (KEGG) |
| C18 | 17 | 1.45522E-39 | muscle filament sliding (BP) |
|  | 11 | 7.45289E-22 | structural constituent of muscle (MF) |
|  | 6 | 2.7683E-15 | troponin complex (CC) |
|  | 9 | 1.50623E-13 | Pancreatic secretion (KEGG) |
| C20 | 41 | 1.5245E-12 | regulation of transcription, DNA-dependent (BP) |
|  | 54 | 3.10885E-14 | DNA binding (MF) |
|  | 119 | 3.50954E-23 | nucleus (CC) |
|  | 12 | 8.59441E-6 | Focal adhesion (KEGG) |
| C28 | 55 | 1.68607E-40 | gene expression (BP) |
|  | 23 | 1.82664E-17 | structural constituent of ribosome (MF) |
|  | 28 | 3.75896E-25 | extracellular matrix (CC) |
|  | 22 | 2.67755E-22 | Ribosome (KEGG) |
| Only significantly enriched functional categories (corrected *P*-value<10^-20^) are presented. The columns of the table summarize the total sizes of the cluster (numbers in parentheses), the number of annotated genes in the cluster, the *P*-value after FDR correction, and the GO categories associated with the cluster. | | | |
