# Supplementary material for: Identifying Subspace Gene Clusters from Microarray Data Using Low-Rank Representation
Source: PLoS One. 2013 Mar 19;8(3):e59377. doi: 10.1371/journal.pone.0059377 (PMC3602020; doi:10.1371/journal.pone.0059377)
Supplement: Table S8 — The most enriched categories of modular enrichment in each gene clusters uncovered by K-means clustering from normal human tissue dataset. (DOC) [file pone.0059377.s008.doc]

Table S8. The most enriched categories of modular enrichment in each gene clusters uncovered by *K*-means clustering from normal human tissue dataset.

| **Cluster** | **No. of genes with**  **in functional category** | **Major GO categories** | **Corrected *P*-value** |
| --- | --- | --- | --- |
| C1(86genes) | 11 | positive regulation of transcription, DNA-dependent | 1.9095E-5 |
| C2(99genes) | 8 | integrin binding | 3.31187E-9 |
| C3(159genes) | 6 | protein transporter activity | 5.56802E-5 |
| C4(144genes) | 11 | protein phosphorylation | 1.37482E-4 |
| C5(330genes) | 4 | nuclear mRNA splicing, via splicesome | 2.12067E-2 |
| C6(514genes) | 6 | receptor activity | 1.61547E-4 |
| C7(109genes) | 3 | heart development | 1.67752E-3 |
| C8(306genes) | 4 | signal transducer activity | 1.6991E-5 |
| C9(257genes) | 4 | transcription factor Activity | 5.94375E-4 |
| C10(100genes) | 4 | proteolysis | 1.99602E-5 |
| C11(92genes) | 3 | ion transport | 2.22585E-6 |
| C12(90genes) | 11 | cornified envelope | 6.72004E-22 |
| C13(231genes) | 3 | axonal fasciculation | 4.23035E-6 |
| C14(586genes) | 8 | integral to membrane | 3.48967E-3 |
| C15(258genes) | 11 | signal transduction | 2.53629E-6 |
| C16(202genes) | 3 | G-protein coupled receptor activity | 2.09912E-3 |
| C17(144genes) | 14 | cytoplasm | 5.26957E-4 |
| C18(243genes) | 6 | nuclear mRNA splicing, via spliceosome | 1.7626E-11 |
| C19(116genes) | 7 | translational elongation | 1.39231E-14 |
| C20(161genes) | 5 | respiratory gaseous exchange | 4.24905E-6 |
| C21(181genes) | 10 | structural constituent of muscle | 2.03117E-16 |
| C22(227genes) | 5 | positive regulation of transcription, DNA-dependent | 8.40019E-5 |
| C23(185genes) | 20 | extracellular region | 1.50459E-8 |
| C24(239genes) | 31 | extracellular region | 4.69045E-19 |
| C25(202genes) | 9 | melanosome | 1.08518E-7 |
| C26(127genes) | 8 | plasma membrane | 2.87415E-5 |
| C27(78genes) | 6 | female pregnancy | 2.41217E-8 |
| C28(95genes) | 3 | nucleotide binding | 4.4539E-4 |
| C29(187genes) | 4 | negative regulation of cell proliferation | 6.20954E-3 |
| C30(165genes) | 21 | mitochondrion | 1.12244E-11 |
| The columns of the table summarize the total sizes of the cluster (numbers in parentheses), the number of genes annotated in the cluster, the GO categories associated with the cluster, and the *P*-value after FDR correction. | | | |
