# Supplementary material for: Identifying Subspace Gene Clusters from Microarray Data Using Low-Rank Representation
Source: PLoS One. 2013 Mar 19;8(3):e59377. doi: 10.1371/journal.pone.0059377 (PMC3602020; doi:10.1371/journal.pone.0059377)
Supplement: Table S9 — The most enriched categories of modular enrichment in each gene clusters uncovered by GPCA from normal human tissue dataset. (DOC) [file pone.0059377.s009.doc]

Table S9. The most enriched categories of modular enrichment in each gene clusters uncovered by GPCA from normal human tissue dataset.

| **Cluster** | **No. of genes with**  **in functional category** | **Major GO categories** | **Corrected *P*-value** |
| --- | --- | --- | --- |
| C1(184genes) | 64 | protein binding | 4.52251E-11 |
| C2(210genes) | 5 | plasma membrane | 7.85399E-7 |
| C3(201genes) | 88 | protein binding | 7.60396E-24 |
| C4(188genes) | 44 | extracellular region | 4.12438E-13 |
| C5(210genes) | 14 | heart development | 3.39229E-10 |
| C6(195genes) | 22 | receptor activity | 2.85758E-11 |
| C7(185genes) | 28 | nucleoplasm | 8.01479E-12 |
| C8(170genes) | 5 | structural constituent of cytoskeleton | 2.43578E-6 |
| C9(186genes) | 16 | plasma membrane | 1.16712E-9 |
| C10(188genes) | 15 | Cytokine-cytokine receptor interaction | 6.06473E-9 |
| C11(195genes) | 4 | response to drug | 1.01554E-5 |
| C12(193genes) | 21 | signal transduction | 3.20421E-4 |
| C13(279genes) | 31 | immune respense | 8.45477E-19 |
| C14(63genes) | 23 | epidermis development | 2.31613E-43 |
| C15(210genes) | 27 | regulation of transcription, DNA-dependent | 3.11486E-4 |
| C16(172genes) | 18 | apoptotic process | 4.3426E-7 |
| C17(196genes) | 27 | regulation of transcription, DNA-dependent | 1.40408E-4 |
| C18(206genes) | 14 | synaptic transmission | 2.57874E-5 |
| C19(208genes) | 22 | multicellular organismal development | 2.15776E-5 |
| C20(252genes) | 18 | signal transduction | 3.59629E-2 |
| C21(353genes) | 45 | blood coagulation | 1.90068E-27 |
| C22(170genes) | 21 | cellular protein metabolic process | 5.09653E-16 |
| C23(206genes) | 20 | transmembrane transport | 2.10609E-6 |
| C24(171genes) | 8 | respiratory electron transport chain | 1.15002E-5 |
| C25(248genes) | 36 | signal transduction | 4.26351E-10 |
| C26(155genes) | 14 | cellular protein metabolic process | 1.76178E-8 |
| C27(207genes) | 15 | multicellular organismal development | 2.01236E-2 |
| C28(198genes) | 14 | negative regulation of cell proliferation | 4.32747E-6 |
| C29(220genes) | 34 | signal transduction | 3.0251E-10 |
| C30(193genes) | 10 | response to organic cyclic compound | 9.021156E-7 |
| The columns of the table summarize the total sizes of the cluster (numbers in parentheses), the number of genes annotated in the cluster, the GO categories associated with the cluster, and the *P*-value after FDR correction. | | | |
