# Supplementary material for: Identifying Subspace Gene Clusters from Microarray Data Using Low-Rank Representation
Source: PLoS One. 2013 Mar 19;8(3):e59377. doi: 10.1371/journal.pone.0059377 (PMC3602020; doi:10.1371/journal.pone.0059377)
Supplement: File S1 — The MATLAB code of Low-Rank Representation is supplied in this Supplementary File. (DOC) [file pone.0059377.s011.doc]

clear;

clc;

[yeastvalues,Genes]=xlsread('yeastdataset');

A=knnimpute(yeastvalues);

x=mapstd(A',0,1)';

X=x';

%%%%%%%%%%%%%%%%%%%%%%%%%%

K=30;

rs = [];

lambda=0.1;

[Z,E] = solve_lrr(X,X,lambda);

%% solve_lrr can be downloaded from http://sites.google.com/site/guangcanliu/

L = abs(Z)+abs(Z');

disp('Perfoming NCut ...');

idx = clu_ncut(L,K);

%% clu_ncut can be downloaded from http://sites.google.com/site/guangcanliu/

rs = [rs,rank(Z,1e-3*norm(Z,2))];

for i=1:K

Cluster{1,i}=Genes(idx==i,:);

SUCCESS=xlswrite('E:\lrr\results\Cluster_lrr', Cluster{1,i}, i);

end
